# Understanding the Flight of the Bumblebee

**DOI:** 10.1371/journal.pbio.1001391

**Published:** 2012-09-20

**Authors:** Robin Meadows

**Affiliations:** Freelance Science Writer, Fairfield, California, United States of America

**Figure:**
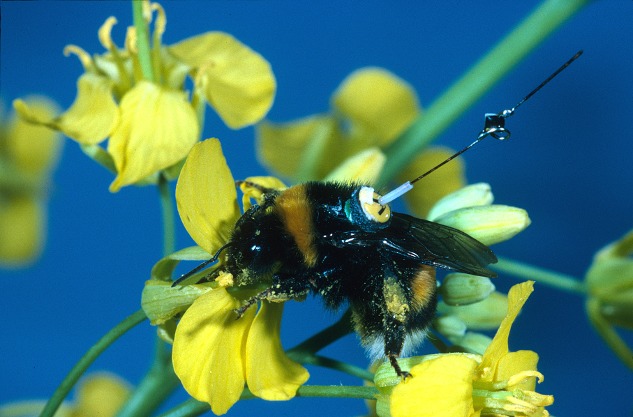
A bumblebee (*Bombus terrestris*) worker with a transponder attached to its back, visiting an oilseed rape flower. (Tracking bees with radar shows how they find an optimal route between multiple flowers.) Image credit: Andrew Martin.

Bumblebees are remarkable navigators. While their flight paths may look scattered to the casual eye, all that buzzing about is anything but random. Like the travelling salesman in the famous mathematical problem of how to take the shortest path along multiple stops, bumblebees quickly find efficient routes among flowers. And once they find a good route, they stick to it. The same goes for other animals from hummingbirds to bats to primates that depend on patchy resources such as nectar and fruit. Perhaps this is not such a surprising feat for animals with relatively high brain power. But how do bumblebees, with their tiny brains, manage it? As new research in this issue of *PLOS Biology* by Lars Chittka and colleagues shows, a simple strategy may be enough for a real-world solution to this complex problem.

For computers, solving the travelling salesman problem means methodically calculating and comparing the lengths of all possible routes. But such an exhaustive approach isn't feasible in practice, and indeed animals can find a near-optimal foraging route, or trapline, without trying them all. Determining exactly how they do this, however, has been stymied by the difficulties of tracking animals as they forage in the wild. Chittka and colleagues got around this problem by tracking bumblebees (*Bombus terrestris*) on five artificial flowers set in a mown pasture. The “flowers” had landing platforms with drops of sucrose in the middle, and were fitted with motion-triggered webcams.

To keep the bees' focus on the artificial flowers, the experiments were done in October, when natural sources of nectar and pollen were scarce. To make the bees want to find all five flowers, each sucrose drop was only enough to fill one-fifth of a bumblebee's crop. And to keep the bees from finding one foraging site from another visually, the flowers were arranged in a pentagon that was 50 m on each side, which is more than three times as far as bumblebees can see them.

The researchers released bees individually from a nest box that was about 60 m from the nearest flower, and used the webcams to track the sequence of flower visits during consecutive foraging bouts. The bees found the closest flowers first and added new flowers during subsequent bouts. With experience, they repeated segments of the visitation sequence that shortened the overall route while abandoning those that did not. Traplines linking all five flowers in a short route were established after an average of 26 foraging bouts, which entailed trying only about 20 of the 120 possible routes.

In addition, the researchers fitted five bees with transponders and tracked them with radar as they developed traplines. This revealed that flight paths between trapline segments were relatively straight and that between their first and last bouts, bees cut their total travel distance by 80% (from 1,953 to 458 m). In contrast to computers, bees did not find the absolute shortest route of 312 m even in this simple experimental arrangement. But they came very close, especially considering that they explored only a small fraction of the possible routes, and established traplines relatively rapidly. This tradeoff between perfection and speed highlights the differences between mathematical and biological solutions to the travelling salesman problem.

How do bees develop such efficient routes so fast? The researchers assessed three possibilities—that bees optimize foraging routes by visiting flowers in the order of discovery, by shuffling them randomly, or by visiting those that are closest together—but found that the first two failed to fit their observations while the third did not fully explain them.

Rather, the researchers propose that bees optimize foraging routes through trial and error, combining exploration with learning from previous bouts to progressively adjust their routes as they find shorter paths. Based on the bees' movements during trapline establishment, the researchers developed a model linking experience to the likelihood of visiting particular flowers. Bees are well-known to be able to compute and memorize distances between locations, and the model assumes that they remember the length of the shortest route so far, compare it to the length of the current route, and then choose the shorter of the two. Over time, choosing the more efficient route favors shorter segments over longer ones. The model is a good fit with the researchers' observations, predicting, for example, that bees will develop and stick to optimal routes in 20–25 bouts.

Besides shedding light on how bees develop traplines, this work suggests that small-brained animals can use simple methods to solve complex routing problems without the need for cognitive maps of spatial relationships, as has been suggested. It remains to be seen whether big-brained animals can also develop traplines with such elementary tools. But if so, that would free up their brain power for other tasks.

**Lihoreau M, Raine NE, Reynolds AM, Stelzer RJ, Lim KS, et al. (2012) Radar Tracking and Motion-Sensitive Cameras on Flowers Reveal the Development of Pollinator Multi-Destination Routes over Large Spatial Scales. doi:10.1371/journal.pbio.1001392.**

